# Antioxidant, Antimutagenic, Tanning and Calpain Induction Activities of Methanolic Extract of Tunisian Plant (*Moricandia Arvensis*)

**Published:** 2017

**Authors:** Ines Skandrani, Ludovik Leloup, Herve Kovacic, Marie-Geneviève Dijoux-Franca, Kamel Ghedira, Leila Chekir Ghedira

**Affiliations:** a *Laboratory of Molecular and Cellular Biology, Faculty of Dental Medecine of Monastir, Tunisia, *; b *Unit of Pharmacognosy/Molecular Biology 99/UR/07-03, Faculty of Pharmacy of Monastir, Tunisia, *; c *University of Aix-Marseille, INSERM U911 (CRO ), 27 boulevard Jean Moulin, 13385 Marseille, France, *; d *Laboratory of Botanic, Pharmacognosy and Phytotherapy, Institut of Pharmaceutical and Biological Sciences, Faculty of Pharmacy of Lyon, France.*

**Keywords:** MeOHL extract, calpain, melanin, ROS, TA104

## Abstract

In this study, we investigate the potential of *Moricandia arvensis *methanol leaf extract (MeOHL) on calpain activity, melanin biosynthesis and DNA mutagenicity. Cytotoxic effect and measurement of reactive oxygen species (ROS) induced by lucigenin in colorectal cells (BE) were also determined. In addition, the chemical analysis of the extract was also studied and the chemical profile illustrates its content in para-hydroxybenzoic acid (*p*-HBA), a glycosylated kampferol (GK), a glycosylated kampferol with Rhamnose (GKR) and 19 amino acids (AAs). Our results showed that MeOHL extract enhanced a significant cytotoxic (max of IP = 89.23%) and antioxidant (max of IP=100%) activities. Furthermore, the tested extract stimulated calpain activity and significantly increased the production of intra (46 µg/mL cells) and extracellular melanin content (12.5µg/mL). Using Ames assay, the extract exhibits a significant inhibition of mutagenicity induced by methy-methane-sulfonate (MMS) (76.32%) as well as 2-aminoanthracene (2-AA) (91%) in the *Salmonella thyphimurium* TA104 assay system.

## Introduction

Epidemiological studies have reported a strong relationship between the consumption of cruciferous vegetables and reduced cancer risk (1). *Moricandia arvensis* (*M.arvensis*) locally called “Kromb ejmel” belongs to the crucifer family. This medicinal plant is used in Tunisia in traditional cooking, and a decoction of leaves and stems was employed in the treatment of syphilis, a sexually transmitted disease, and scorbut (2). The chemical constituents of *M.arvensis* studied include flavonoids, tannins, coumarins, terpenes, sterols, and iridoids from leaves and roots. In addition, various extracts have been tested for different biological activities (2-5). Besides, the reported biological activities, there is one publication (6) which identified pure compounds (glucosinolates) from the flowers. The biological approaches to validate *M. arvensis* bioactivities however were limited and only one phytochemical study was carried out on this plant (6). 

There is evidence that several plant antioxidants have anticarcinogenic effects and have also been shown to inhibit cancer cell proliferation *in-vitro* (7). The main cause of the formation of cancer cells is ROS, which have the potential to damage cell components and may help to induce the apoptosis (8). Among the most important proteins in the apoptotic phenomenon are the calpains, which are recently considered as the key of apoptosis and are involved in many physiological and pathological processes. However, control of their activity by limiting their functions has caught the attention of drug development (9). Therefore, the present study examines the inhibitory effects of methanol extract (MeOHL) obtained from the leaves of *M.arvensis*, on the proliferation of cancerous colorectal cell lines (BE), and calpain activity. Moreover, its ability to inhibit the formation of ROS in the BE cells was also investigated.

Recently, much attention focused on cosmetology on the application of naturally occurring drugs. As most people have become aware of the risks associated with UV exposure, self-tanning products now represent a continuously growing segment of the cosmetic market (10). The most common hypopigmentant skin disorder is vitiligo, which is a depigmentation disease (11) characterized by the destruction of melanocytes (12). Therefore, in order to develop a new tanning agent, MeOHL extract was also evaluated for melanogenesis stimulatory activity, mutagenicity and antimutagenic effect.

## Experimental

*Plant Material*

Leaves of *M.arvensis* were collected in Gafsa (Oued Ghezran) in the southern region of Tunisia (December 2012). *M.arvensis* subsp. *eu-arvensis *is a medicinal plant identified by Pr. M. Cheieb (Department of Botany, Faculty of Sciences, Sfax, Tunisia). 

*Preparation of Plant Extract*

The leaves of *M.arvensis* were dried at room temperature, ground and then stored in our laboratory for preparing extracts. Using soxhlet, the plant was degreased with petroleum ether and choloroformic solvents. Then, methanolic solvent was used to obtain the various polar molecules thereby forming the methanol extract (MeOHL) which was concentrated to dryness and the residue was kept at 4 °C.

*Fractionation and purification of the MeOH extract from the leaves of Moricandia arvensis*

**Figure 1:**
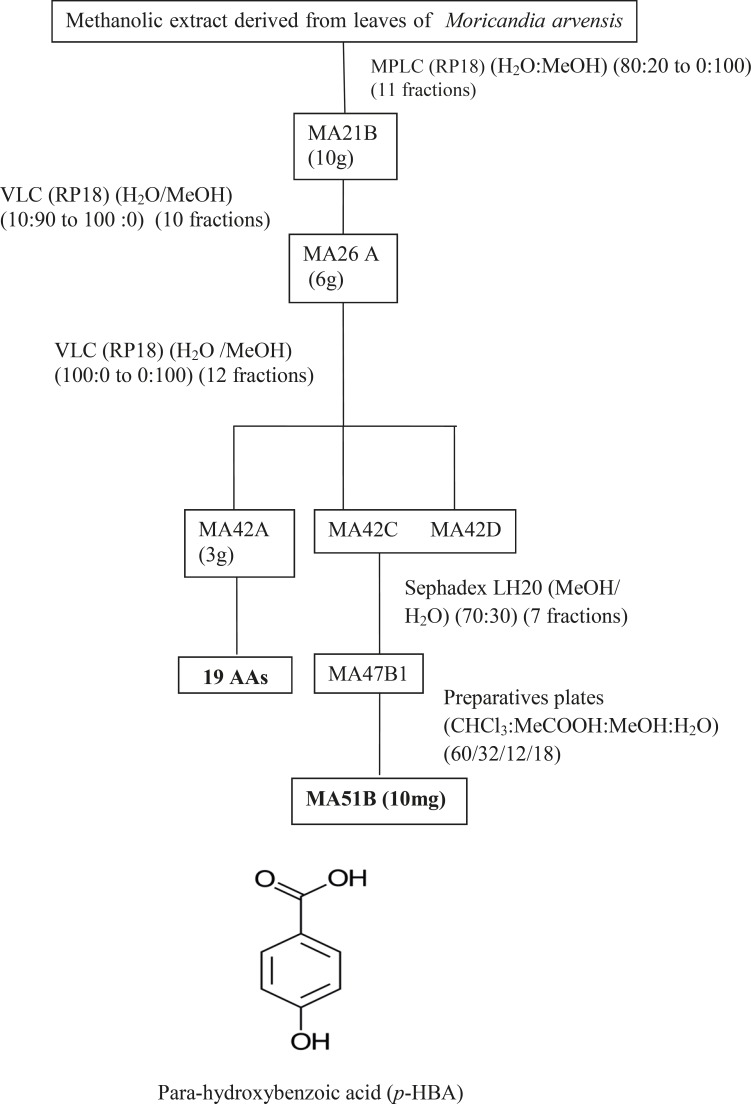
Purification of MA51B resulted from fractionation of MeOHL extract.

**Figure 2:**
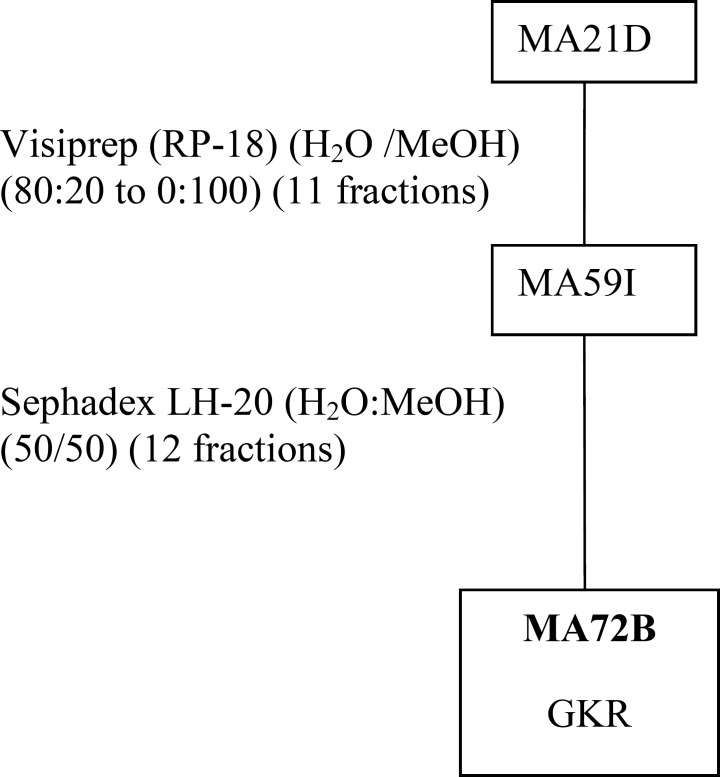
Purification of MA72B obtained from fractionation of MeOHL extract.

**Figure 3:**
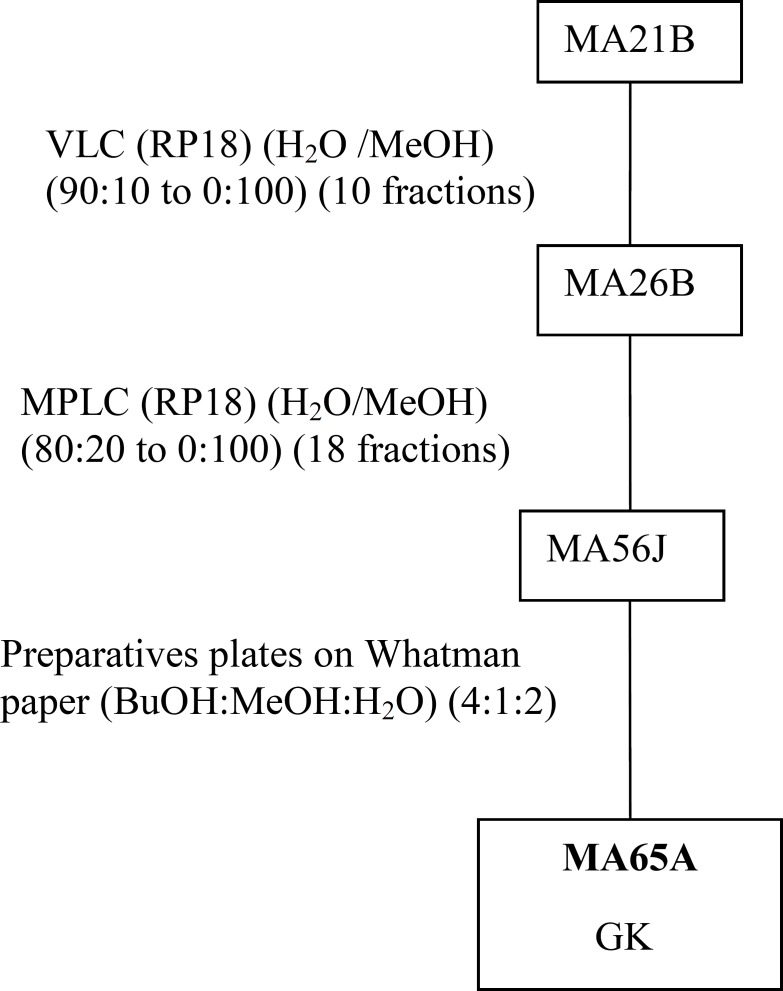
Purification of MA65A obtained from fractionation of MeOHL extract.MA : *Moricandia arvensis*; VLC: Vacum liquid chromatography; MPLC : medium pressure liquid chromatography; GK: Glycosylated Keampferol

MeOHL extract was fractionated by a reverse phase (RP-18) by MPLC (Medium Pressure Liquid Chromatography) using MeOH: H2O as eluent solvent system to afford 11 different fractions (21A-21K).The mixture of MA42C (44.7 mg) and MA42D (44.7 mg) fractions were separated on a Sephadex LH-20 to obtain 7 fractions (47A- 47D), eluted with a mixture of H_2_O-MeOH (30:70). The resultant MA47B1 fraction (28.5 mg), after separation on preparative plates using CHCl_3_-MeOH-H_2_O-MeCOOH (60-32-12-8), led to obtain MA51B compound (10 mg) (Figure 1). The MA21B fraction (7g) was subjected to separation on VLC (Vacuum Liquid Chromatography) eluted with MeOH-H_2_O solvent system with gradual increasing with MeOH (10:90 to 100:0) and 10 fractions (26A-26J) were collected. MA26A fraction (6g), after separation on reversed phase (RP-18) using MeOH-H_2_O (100:0 to 0:100), gives 12 different fractions (MA42A to MA42L). The resultant MA42A fraction (3g) representing 50% of MA26A and obtained with 100% water is a mixture of 19 AAs (Table 1) identified by HPLC with UV detection. Quantitative HPLC analysis of AAs is achievable through the use of two reagents, i.e Acid O-Phthalaldehyde (OPA) and 9-Fluorenylmethyl Chloroformate (FMOC). The obtained MA21D fraction (129 mg) was chromatographed by SPE (Solid Phase Extraction) on RP-18 using H_2_O:MeOH (80:20 to 0:100) as a solvent system to afford 11 fractions (59A-59K). The MA59I fraction (12.1 mg) was fractionated on Sephadex LH-20 with a mixture solvent of H_2_O:MeOH (60/40) which allowed us to afford the pure compound MA72B (1.2 mg) (Figure 2). The MA26B fraction (1.235 g) underwent a first fractionation by MPLC on a reverse phase chromatography (RP-18) eluted with H_2_O:MeOH with gradient increasing of the MeOH (80:20 to 0: 100) and 18 fractions (56A-56Q) were collected. The MA56J fraction (35 mg) was chromatographed on a Whatman paper using an BuOH:MeOH:H_2_O (4:1:2) solvent system to afford compound 3 (MA65A) (1 mg) (Figure 3).

**Table 1:** Nuclear magnetic resonance of MA51B (*p*-HBA).

				**Literature (**Velandia *et al*., 2002**)**	
	^1^ **H**		^13^ **C**	^1^ **H**	^13^ **C**
N°	δ (ppm)^a^	(Hz) J	δ (ppm) a	δ (ppm)^a^	δ (ppm)^a^
1	-	-	176.2	-	167.7
2	-	-	112.3	-	122.0
3/7	8.78	nd	122.1	7.71	132.9
4/6	7.46	nd	119.4	6.68	116.3
5	-	-	150	-	163.5

a : DMSO-D6

*Cell culture and cell viability assay*

The human colon cancer cell line (BE) was maintained in DMEM (Dulbecco’s modified Eagle’s medium) medium supplemented with 10% fetal bovine serum and 1% L-glutamine (from Sigma Cell Culture, Courtaboeuf, France), in humidified incubator at 37°C with its atmosphere enriched with 5% CO_2_. To control the impact of the tested concentrations of MeOHL extract (200, 400, 600 or 800 µg/mL) on cell viability, we estimated viability of BE cells by the 3-(4, 5-dimethylthiazol-2-yl)-2, 5-diphenyltetrazolium bromide (MTT) assay, which is based on the cleavage of a tetrazolium salt by mitochondrial dehydrogenase in viable cells. Cells were seeded in 96-well microtiterplates; 24 h later, the test samples were added in serial dilutions before incubating the plates for 48 h. Cells were washed once before adding 10 μL of PBS containing 5 mg/mL MTT in 100 µL of medium. After two hours of incubation at 37°C, the medium was discarded, and the formazan blue formed in the cells was dissolved by adding 100 μL DMSO. Negative control (NC) without the tested extract was prepared in the same manner. Optical density (OD) was measured at 570 nm. Data were obtained from triplicate wells. Cell cytotoxicity was calculated as follows:

 Cell cytotoxicity (%) = (1- (OD of treated cells /OD of NC) × 100).

 NC: Negative control (untreated cells).

*Quantification of calpain activity*

BE cells were treated with different concentrations of MeOHL extract (200, 400 and 600 µg/mL) for 2h, 4h and 6h. The global calpain activity was observed and quantified after 30 min in darkness using 7-amino-4-chloromethylcoumarin (t-BOC-LM-CMAC) (25 µM). This molecule enters passively in cells and becomes fluorescent after calpain cleavage. The plate containing the various tested concentrations was observed under a fluorescent microscope (excitation 329 nm, emission 409 nm). The inhibition percentage of calpain activity was ultimately calculated using the following formula:

Inhibition of calpain activity (%) = (1− (FS/FC)) × 100 

FS: Fluorescence of treated BE cells with the different concentrations of MeOHL extract.

FC: Fluorescence of NC (untreated cells).

*Measurement of ROS production*

ROS production was measured as reported by Shen *et al*. (13) with some modifications. Briefly, BE suspension (5x10^4^ cells/well) were seeded in white 96-well, flat-bottom microplates. After 24 h of incubation, different concentrations of MeOHL extract (200 400 and 600 µg/mL) were added for the three time periods of incubation (2h, 4h and 6h). The cells were triggered by adding a mixture of lucigenin (50 µM) and NADPH (4 mM). Chemiluminescence was monitored every minute for 45 min, using a microplate luminometer reader. The inhibition percentage of ROS production was calculated as follows:

 The inhibition percentage of ROS production (%) = (1- (OD of treated cells /OD of control cells) × 100). 

*Measurement of melanin content on B16-F0 cells*

B16-F0 cells (2×10^5^) seeded with 10 mL of culture medium and incubated for 24h were treated with MeOHL extract for 48h. To determine the amount of intracellular melanin, adherent cells were detached by incubation in trypsin (2.5%) and ethylenediamine tetraacetic acid (0.11%). Tubes containing 10^6 ^cells were prepared and solubilized in 1 mL of Triton X100 (0.1%), in order to quantify extracellular melanin. Spectrophotometric absorbance was performed at 475 nm and the absorbance was compared to a standard curve of known concentrations of synthetic melanin. α-Melanocyte-stimulating hormone (α-MSH) was used as a positive control (PC).

*Bacterial Tester Strain and S9 fraction*

TA104 (*hisG428, rfa, ∆uvrB, pKM101*) strain of *Salmonella typhimurium* (*S.typhimurium*) was kindly supplied by Dr. I. Felzen, Universidade do Estado do Rio de Janeiro (UERJ, Brazil). This strain contains an ochre mutation AT base pairs at the hisG428 mutant site which can be reverted by mutagens causing oxidative damage (14). The S9 fractions were prepared from livers of rats according to the method described by Maron and Ames (15).

*Mutagenicity assay in vitro*

This test is used to examine whether MeOHL extract is capable of inducing a mutation through oxidative damage, on a gene of *S. typhimurium *TA104. The tested strain has a mutation, making it unable to grow on a medium without histidine (*his*^-^). If the tested extract has a mutagenic effect, it induces a reversion of this mutation and gives bacteria the ability to grow on medium without histidine (*his*^+^). In addition to the histidine mutation, the strains contain other mutations that increase their sensitivity to mutagens. As described by Maron and Ames (15), the experiments were performed with and without an exogenous metabolic system (S9 fraction in S9 mix). The revertant bacterial colonies of each plate were counted and compared to the number of spontaneous revertants (SR) of negative control (NC: untreated strain with tested extract) and the number of revertants of positive controls (RPC) (the direct mutagen: MMS and the indirect mutagen: 2-AA). The extract was considered mutagenic if the number of revertants of MeOHL extract (R MeOHL) per plate was doubled compared to the SR of the NC. 

*Antimutagenicity assay in-vitro*

A modified procedure described by Ferrer *et al*. (16) was employed to determine the effect of MeOHL extract on MMS and 2-AA induced mutagenicity. The inhibition percentage of mutagenicity (%) was calculated by the following formula: 

Inhibition percentage (%) = [1− (RMeOHL - SR)/(RPC - SR)] × 100.

Where: RMeOHL is the number of revertants on MeOHL extract plates,

 RPC is the number of revertants on positive mutagen control plates. 

 SR is the number of spontaneous revertants on negative control plates.

*Statistical analysis*

All data were expressed as mean (± SD) and compared using a Student’s *t*-test. Statistical significance was assigned at *p* values < 0.05. All data were analyzed using SPSS 11.0 software (SPSS INC; Illinois, USA). 

## Results

*Purification of the MeOHL extract from leaves of Moricandia arvensis and identification of purified compounds*

The isolation of molecules of the MeOHL extract was particularly difficult due to the presence of tannins. These compounds with high molecular weight make the isolation of pure molecules extremely difficult. Therefore, in this preliminary work we focused on testing several chromatographic methods in combination with various solvent systems, to develop a fractionation system which facilitates the separation of tannins from the other compounds. Alternating adsorption chromatography (in normal and reverse phases) and exclusion partially addresses this problem. However, many purification steps are needed, making the final yield of pure compound very low. Thus, only three pure compounds could be isolated and only one was identified with precision. Therefore, the various spectral data and comparison with the literature allowed us to confirm that MA51B is Para-Hydroxybenzoic Acid (*p*-HBA) (Table 1) (17). The structural identification of compound was established using spectroscopic analysis, especially, NMR spectra. Comparing with the standard range, we have also quantified 19 AAs and given their concentration (Table 2). The low mass of MA72B (1.2 mg) (Figure 2) did not achieve the complete identification of this compound, which is a Kaempferol (flavonol) with Glucose and Rhamnose (GKR). Similarly, we could not complete identification of (MA65A) (1 mg) (Figure 3), but we could determine the general structure which is a glycosylated kaempferol (GK).

**Table 2:** Quantification of amino-acids from MA42A fraction.

** AAs **	**Abbreviation**	** Concentrations (mg/L)**
Aspartic acid	Asp	11.69
Glutamic acid	Glu	24.55
Asparagine	Asn	180.66
Serine	Ser	63.52
Glutamine	Gln	0.00
Histidine	His	33.82
Glycine	Gly	30.88
Threonine	Thr	30.23
Arginine	Arg	84.73
Alanine	Ala	133.40
Tyrosine	Tyr	6.10
Cystine	Cys	75.56
Valine	Val	74.99
Methionine	Met	15.81
Tryptophane	Trp	13.83
Phenylalanine	Phe	13.65
Isoleucine	Ile	17.50
Leucine	Leu	21.54
Lysine	Lys	26.50
Proline	Pro	677.65

The bold caracter: the essential amino acids.

AAs : Amino-acids

*Cell viability assay*

**Figure 4:**
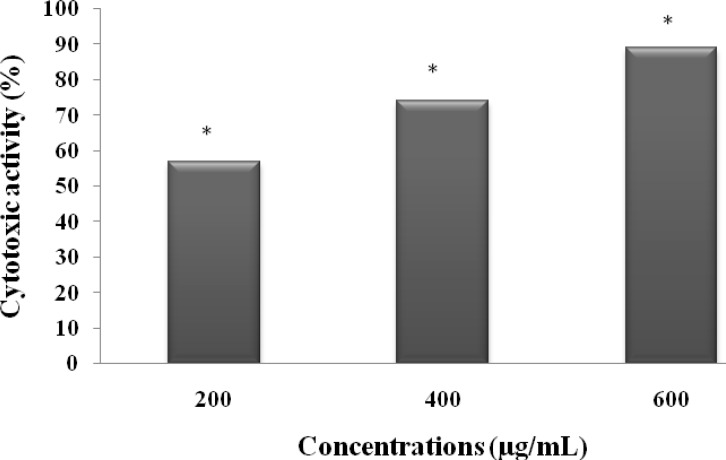
Cytotoxic effect of MeOHL extract on colon cancer (BE) cells. Cells were treated with different concentrations of MeOHL extract for 48 h, and cytotoxic effects were determined by MTT assay. The values are represented as the percentage of cell cytotoxicity. Values represent mean ± SE of three independent experiments

The potential cytotoxic effect of MeOHL extract on a human colorectal cell line (BE) was investigated. Figure 4 showed that MeOHL extract was able to induce cytotoxicity in BE cells in a dose dependent manner. After 48h of incubation, the lowest concentration (200 µg/mL) was able to reduce cell proliferation by 56.84%. This anti-proliferative effect increase by 89.23% when colorectal cells were incubated with the highest concentration (800 µg/mL).

*Calpain activity*

**Figure 5:**
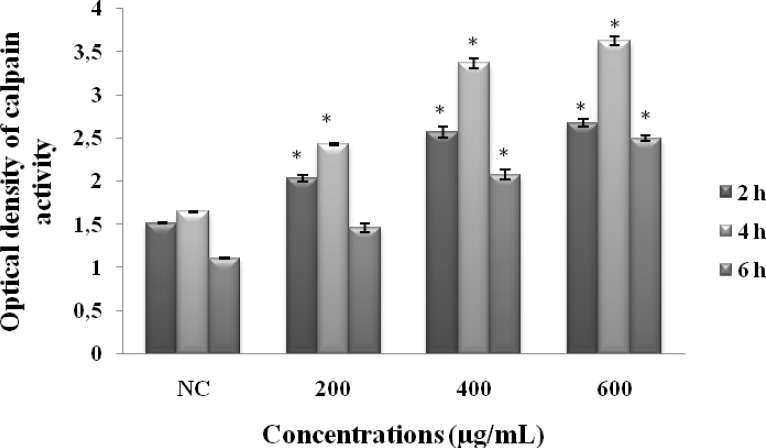
Effect of MeOHL extract on the calpain activity using colorectal cancer (BE) cells. Cells were treated with different concentrations of the extract for 2 h, 4 h and 6 h, and calpain activity was determined by tBOC-LM-CMAC assay. Data represent the mean ± SE of 3 experiments, each conducted in triplicate. (*) *p* < 0.05 means a significant difference between the untreated and treated cells

Compared to NC, our results showed that MeOHL extract stimulated calpain activity at a dose dependent manner (after 2h of incubation, OD= 2.68 at 600 µg/mL compared to NC: OD= 1.52). This effect undergoes a slight decrease when we increase the time of incubation (after 6h of incubation, OD= 2.5 at 600 µg/mL compared to NC: OD= 1.11) (Figure 5). 

*Measurement of ROS production in-vitro*

**Figure 6:**
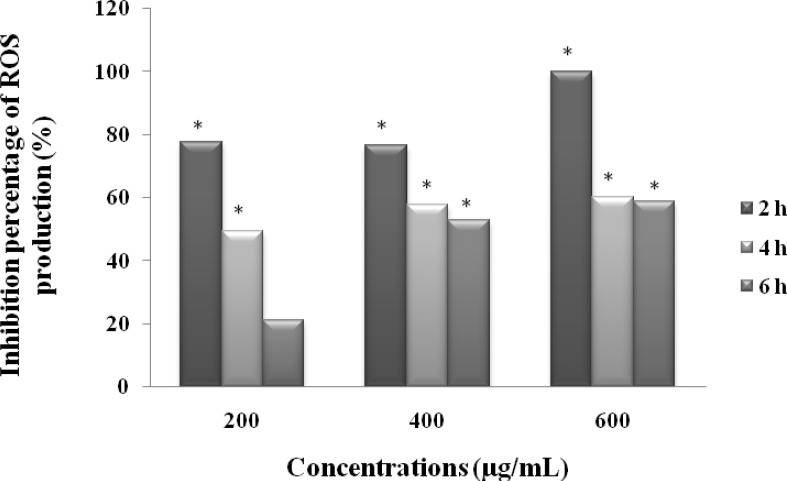
Effect of MeOHL extract from *M.arvensis* on the inhibition of lucigenin-induced ROS production in human cancerous colorectal cells (BE). The values are represented as the percentage of ROS production inhibition. Data represent the mean ± SE of 3 experiments, each conducted in triplicate. (*) *p* < 0.05 means a significant difference between the untreated and treated cells

To examine whether MeOHL extract could prevent ROS production, we set up an *in vitro* method with lucigenin-amplified chemiluminescence, measuring thus the extracellular ROS production in BE cell line. The results summarized in Figure 6 show that MeOHL extract has a potent antioxidant activity in a dose dependent manner. Indeed, at the lowest concentration 200 µg/mL, MeOHL extract inhibits lucigenin to induce ROS production by 77.67%, after 2 h of incubation. This effect increases when increasing the amount of extract, reaching thus 100% at a concentration of 600 µg/mL. After 6 h of incubation, the antioxidant effect decreases with the different tested concentrations to reach a value of 58.82% at a concentration of 600 µg/mL. 

*Measurement of melanin content on B16-F0 cells*

**Figure 7:**
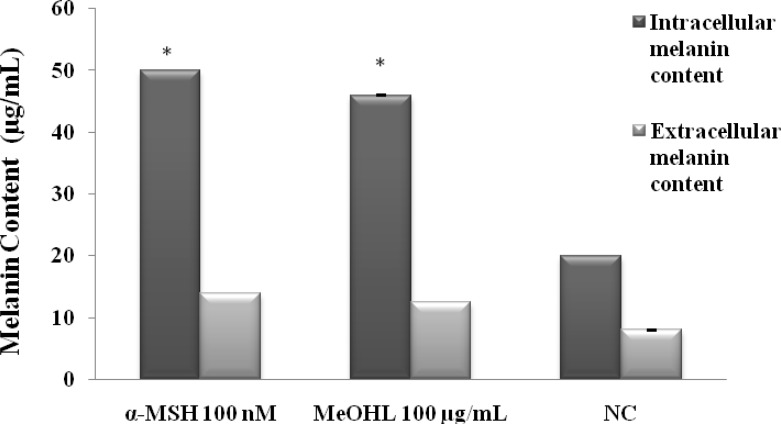
Effect of MeOHL extract (100 µg/mL) on melanin content in B16-F0 cells after 48 h of incubation. MeOHL: Methanol leaf extract. α-MSH: α-Melanocyte-stimulating hormone.  (*) *p *< 0.05 means a significant difference between the treated cells and untreated cells (NC

To investigate the effects of MeOHL extract on melanogenesis, the melanin content of the B16-F0 cells with and without treatment of tested extract were measured. As shown in Figure 7, MeOHL extract significantly increased the production of intra (46 µg/mL cells) and extracellular melanin content (12.5 µg/mL) when compared with untreated cells (20 µg/mL of intracellular melanin content and 8 µg/mL of extracellular melanin content) and with α-MSH (50 µg/mL and 14 µg/mL of intra and extracellular melanin contents respectively). 

*Mutagenicity and antimutagenicity tests in-vitro*

Table 3 shows that MeOHL extract did not induce an increase in the number of revertants in TA104 strain, as well as with or without S9 metabolic system. The addition of MeOHL extract to TA104 tested strain was effective in reduction of the mutagenicity caused by the direct mutagen MMS by 76.32% (at the highest tested dose of 50 µg/plate) (Table 4). Similarly, the tested extract exhibited significant inhibition activity against indirect mutagen 2-AA induced mutagenicity, in a dose dependant manner. The highest inhibition percentage obtained was 91%, when 50 µg/plate of extract was added to the *S. thyphimurium* TA104 assay system. 

**Table 3:** Mutagenic effect of MeOHL extract from *M. arvensis* in *Salmonella typhimurium *TA104 assay system in the presence and absence of exogenous metabolic activation system (S9).

**Extract**	**Dose µg/plate**	**Number of revertants/plate**	**Number of revertants/plate**
		**-S9**	**+S9**
MeOHL extract	100	415±15	497±7
	250	500±11	525±0
	500	479±1	647±1
SR	-	477±12	744±18
PC(MMS)	0.65	3400±31*	**-**
**PC (2-AA)**	**5**	**-**	2130±23*

Positive control (PC); methyl-methane-sulfonate (MMS) (0.65 µg/plate); 2-aminoanthracene (2-AA) (5 µg/plate); SR: Spontaneous revertants; MeOHL (Methanol leaf extract).

(*) p < 0.05 means a significant difference between the revertants of negative control (SR) and the revertants of MeOHL extract.

**Table 4:** Effect of MeOHL extract on the mutagenicity induced by MMS and 2-AA in the *Salmonella typhimurium* TA104 assay system

*Effect of MeOHL extract on the mutagenicity induced by MMS *				*Effect of MeOHL extract on the mutagenicity induced by 2-AA *	
	**Doses** **µg/plate**	**Nb revertants/plate**	**IP (%)**	**Nb revertants/plate**	**IP (%)**
MeOH	5 10 50	849±15* 1922±6* 2173±5*	89.15 52.44 3.89	476±14* 876±23* 668±64*	99.64 75.70 88.15
SR	-	532±15	-	420±10	-
SR+S9	-	-	-	470±9	-
MMS	0 .65	3455± 38	-		-
2-AA	5	-	-	2141±27	-

Positive control (PC); methyl-methane-sulfonate (MMS) (0.65 µg/plate); 2-aminoanthracene (2-AA) (5 µg/plate); Spontaneous revertants (SR). Spontaneous revertants induced after metabolic activation with S9 (SR+S9). Number (Nb). MeOHL (Methanol leaf extract).

(*) p < 0.05 means a significant difference between the revertants of positive control and the revertants of extract.

## Discussion

In this present study, we have shown an important cytotoxicity against the cancerous colorectal cells. This strong antiproliferative activity may be attributed to the presence of specific components, such as polyphenols which are able to alter proliferation in colon cancer cell lines (18). In our preliminary study, we have demonstrated that MeOHL extract contained a high phenolic content (2), which coincides with its cytotoxic activity. Moreover, cell growth inhibitory activity of extract could be, at least partially, the consequence of phenolics presence, such as *p*-HBA and GK found in our extract. It is well–known that this class of natural products can exert potent cytotoxic activity against different cancer cell lines for example Sahpazidou *et al*. (18) have demonstrated cytotoxic activity of polyphenolic extracts from grape stems against colon cancer cells. Morever, Heleno *et al*. (19) have reported the cytotoxicity of *p*-HBA against cancerous cell lines (HCT15). It has already described many flavonoid glycosides with cytotoxic activity for example the kaempferol 3-O-alpha-Lrhamnopyranosyl-(1–>2)-alpha-L-arabinopyranose showed strong cytotoxicity against human small lung cancer cell line A549 and melanoma SK-Mel-2 (20). Likewise, Tofigh *et al*. (21) have found that the orobol-4′-O-glucopyranoside, a flavonoid glycoside isolated from the seed extract of *S. securidaca* showed a potent cytotoxicity against colorectal cancer cells (HT-29 and Caco2). Antunes-Ricardo *et al*. (20) have shown that glycosylation patterns play an important role in cytotoxicity through cell death mechanisms that they can induce. On the other hand, recent researches have shown that a glycolased flavonoid (Kampferol-3-O-glucoside) and *p*-HBA did not induce cytotoxicity in human colon carcinoma cells HCT 116 (22) and against HCT15 (23). Therefore, the inhibitory activity of MeOHL extract against cancer cell growth is due to other phytochemical compounds apart from polyphenols, or there is a synergism between polyphenols and other phytochemical compounds (20) besides *p*-HBA and flavonoid glycosides.

To examine the mechanisms by which cytotoxicity is induced, we tested the effects of MeOHL extract on calpains, which are considered as potential anticancer targets (9). Indeed, our study demonstrates that MeOHL extract stimulated calpain activity in BE cells. Both stimulation of calpain activity and cytotoxic effect by the tested extract may classify it, as an anticancer agent, but further research on its mechanism of action should be conducted. Several chemotherapy drugs activate the ubiquitous calpains causing cell death (9). Antiproliferation and the removal of malignant cells through induction of apoptosis have been proposed as another strategy for cancer prevention (24). Phenolic compounds are postulated to be effective in inducing apoptosis and as anticancer agents. These molecules may act as cancer-blocking agents, preventing the initiation of the carcinogenic process as cancer-suppressing agents and inhibiting cancer promotion and progression (25). Other reports showed that flavonoids and their glycosides were able to produce cell death through the caspases activation (26). Many glycosylated flavonoids have demonstrated cytotoxic and apoptotic effects in different cell lines, but mainly against colon cancer cells because of their high bioavailability in the large intestine where they are cleaved by bacterial enzymes to yield the corresponding aglycones (27). Antunes-Ricardo *et al*. (20) have indicated that the glycosylation pattern affects the percentage of apoptotic colon cancer cells and also Loizzo *et al*. (28) have established that biological activities of flavonoids depends on their glycosylation profile, the type, position and number of sugar moieties. The chemical profile of our extract also showed the presence of AAs which have been described as antitumor agents (29). The consumption of cruciferous vegetables has long been associated with a reduced risk in the occurrence of cancer at various sites, including colon cancer (1).The apoptotic activity of MeOHL extract may also be due to compounds derived from the cruciferous family. However, Sulforaphane as an isothiocyanate compound isolated from broccoli extract, has been shown to induce apoptosis of different types of cancer cells (30) acting thus as a therapeutic and/or preventive agent. Likewise, crucifers contain many bioactive components including flavonoids, polyphenols (4) minerals, vitamins, indole derivative,s and glucosinolates (6) which are among the most studied bioactive compounds associated with cancer protection (31).

Plants rich in antioxidant phytochemicals are important for the prevention of diseases related to ROS such as cancer. For this reason we tested the ability of our extract to reduce or inhibit ROS and we have found that MeOHL extract has a potent antioxidant effect against the ROS induced by lucigenin in BE colorectal cell line. This result indicates an important role for these antioxidants in controlling cancer cell proliferation *in vitro* (32). Nicholas *et al*. (33) have demonstrated that HBAs may be the most significant phenolic antioxidants in many foods indicating they are effective dietary antioxidants. Kanupriya *et al*. (34) have reported that HBA and other phenolic isolated from *Rhodiola sacra* compounds have strong scavenging activities against O_2_^.−^ and OH^.^ radicals. Further, Heleno *et al*. (19) have illustrated that *p*-HBA has antioxidant activities against free radicals. Isovitexin, a glycosyl flavonoid, has an antioxidant activity comparable to those of tocopherol and ascorbic acid (35). Jung *et al*. (36) have showed a potent antioxidant activity of Kaempferol glycosides isolated from *Brassica juncea* and other studies demonstrated that flavanoids like Galanin and Kaempherol protect the cells from oxidative stress by scavenging ROS (34). The antioxidant activity of MeOHL extract may also be attributed to its AAs content. Effectively, Trp has been reported to play an important role in the DPPH radical scavenging activity, perhaps as a hydrogen donor. In addition to the contribution of Trp to OH^•^ scavenging activity, Phe may have also played an important antioxidant role through its aromatic ring which can react with hydroxyl radicals (37). Furthermore, His was considered to be a strong antioxidant AA through its metal chelator effect due to the presence of an imidazole ring. Pownall *et al*. (37) also have proved that the combined fraction formed by Phe, Ile, Leu, and Val exhibited a strong O_2_^•^^-^ scavenging activity, which may results from the hydrophobic nature of AAs. During this study of the antioxidant activity, we have noticed that the most important effect focused occurred after 2 h of incubation and then decreased with increasing incubation time. This phenomenon could be explained by the occurrence of a biphasic reaction between radicals and polyphenols (2). In the first very rapid reaction, the more active compounds scavenge immediately ROS. Then a second reaction affected the products formed together with the less reactive molecules thus explaining the decrease of antioxidant effect after 6 h of incubation.

Melanin is a pigment that determines skin color and is an important defense mechanism against the harmful ultraviolet rays. For medical and cosmetic reasons, the demand for effective hyperpigmentating agents has increased. MeOHL extract was screened for its activity against melanin biosynthesis in B16-F0 mouse melanoma cells and it was found that the extract stimulates melanin production. Therefore, MeOHL extract may play a role in the tanning effect of skin and might hold significant therapeutic value in the prevention or treatment of hypopigmentation and depigmentation disease such as vitiligo (11). It s well known that melanin acts as a photoprotector by absorbing UV sunlight and plays an important role in scavenging ROS (5). Increased melanin production caused by MeOHL extract indicates its potential use in cosmetology and as a photoprotector against UV radiation, and as a tanning product. Tanning or UV-induced melanogenesis offers unparalleled protection against skin cancer. Our extract triggers tanning and stimulates melanogenesis at non cytotoxic doses; this result was in concordance with a few reports such as those found by Won *et al*. (38) which described the stimulation of melanogenesis, by triterpene glycosides without cytotoxicity in B16-F0 melanoma cells suggesting that plant glycosides have a high affinity for cell membranes. Other reports have proved that the stimulatory effect on melanogenesis depends on the type or position of the sugar moiety (39) and glycoside structure (36). Wang *el al*. (40) have showed that *p*-HBA inhibited tyrosinase activity reflecting thereby the inhibitory effect on melanogenesis. Likewise, Lin *et al*. (41) reported that AAs showed a decrease of melanin production in B16-F0 melanoma cells. This could indicate that *p*-HBA and AAs do not have any effect on the stimulation of melanogenesis and that the stimulatory activity found by our extract may be due to molecules other than *p*-HBA and AAs. 

Based on these important biological activities, it is important to know if MeOHL extract has a mutagenic effect and if it is able to prevent DNA against mutagens induced ROS. We showed that MeOHL extract was not mutagenic, either alone or in the presence of S9 and exhibited a significant antimutagenic effect towards 2-AA more than MMS. Considering that TA104 strain is special to detect mutations caused by free radicals (14), the extract may be more efficient in indirectly inhibiting ROS formed during the process of microsomal enzyme activation than directly the ROS induced by MMS. The significant antimutagenic activity is congruent with its strong antioxidant capacity and may be related to its high content of polyphenols ; a relationship that has been demonstrated by other studies for other extracts (42). In fact, MeOHL extract was enriched with phenolic molecules which have been described as antimutagens (2, 3), proving that consumption of the studied extract could be an alternative for reducing mutagenic damage induced by ROS. The antimutagenic properties of *p*-HBA contained in our extract, have been described by Khadem and Marles (43) and Heleno *et al*. (19). Moreover, this antimutagenic activity can also be due to the glycosylated flavonoids which they exert their antimutagenicity effect through their higher number of hydroxyl functions (44). Another important feature was that the carbonyl function at C-4 of the flavonoid nucleus was essential for antimutagenicity (45). Choi *et al*. (46) illustrated that the possible antimutagenic mechanism of flavonoids was demonstrated as inhibitory action on DNA-adduct formation through interaction with microsomal activating enzymes. The antimutagenic activity can also be due to the richness of our extract in AAs. Effectively, Rawat and  Meena (47) have discovered that L-Cys was more potent as antimutagen than vitamin C. The antimutagenic activity of AAs may be due to the role that they play in the synthesis of purines and pyrimidines whereby they could supply the bacteria with correct nucleic acid constituents. For example, Glu may contribute to the amino group on pyrimidines and to N1, of the ring. Ser takes part in the formation of C2, C4, C8, and N7, of purines and the CH_3_ group of thymine. Methionine can act as a methyl donor in the conversion of aminoimidazolecarboxamide to purines (48). It is believed that AAs act as desmutagens, i.e directly on mutagens/their precursors/ their metabolic activation and inactivate them; or they act on the process of mutagenesis or repair DNA damage, resulting in a decrease in mutation frequency. It is worth noting that the amino group of AAs plays a major role in the antimutagenecity by forming N-nitroso compounds. However, the mechanism of this antimutagenic effect could not be explained by a direct effect of the amino group only, because the inhibition varied markedly with the structure of R groups. The SH group in Cys is thought to undergo rapid methylation by trapping methy radicals generated from MMS or 2-AA. Among the AAs, Gly has the simplest structure but was more antimutagen than Asn, Gln, Lys, and some others (49).

## Conclusion

 Overall, it is worth considering that MeOHL extract may have nutritional benefits, not only as antioxidant and antimutagenic compounds, but also as chemopreventive agents against carcinogenic disease. Consumption of MeOHL extract containing AAs, *p*-HBA and glycolysated flavonoids, as a dietary supplement has the potential to provide health benefits via alleviation of radical-induced mutagenicity and cancer. Improved knowledge of its proapoptotic properties could support its rational use as nutraceutical and therapeutic agents. In addition, our studied extract may be a novel melanogenesis stimulator which can be used as a natural product for tanning in cosmetic applications. 
